# D-shaped plastic optical fibre aptasensor for fast thrombin detection in nanomolar range

**DOI:** 10.1038/s41598-019-55248-x

**Published:** 2019-12-10

**Authors:** Nunzio Cennamo, Laura Pasquardini, Francesco Arcadio, Lia E. Vanzetti, Alessandra Maria Bossi, Luigi Zeni

**Affiliations:** 1 Department of Engineering, University of Campania “L. Vanvitelli”, Via Roma 29, Aversa, Italy; 2 Indivenire srl, Via Alla Cascata 56/C, 38123 Trento, Italy; 3 Fondazione Bruno Kessler-CMM-MNF, Via Sommarive 18, Trento, Italy; 4 Department of Biotechnology, University of Verona, Cà Vignal 1, Strada Le Grazie 15, 37134 Verona, Italy

**Keywords:** Applied optics, Optical techniques

## Abstract

The development of optical biosensors for the rapid and costless determination of clinical biomarkers is of paramount importance in medicine. Here we report a fast and low-cost biosensor based on a plasmonic D-shaped plastic optical fibre (POF) sensor derivatized with an aptamer specific for the recognition of thrombin, the target marker of blood homeostasis and coagulation cascade. In particular, we designed a functional interface based on a Self Assembled Monolayer (SAM) composed of short Poly Ethylene Glycol (PEG) chains and biotin-modified PEG thiol in ratio 8:2 mol:mol, these latter serving as baits for the binding of the aptamer through streptavidin-chemistry. The SAM was studied by X-ray Photoelectron Spectroscopy (XPS) analysis, static contact angle (CA), Surface Plasmon Resonance (SPR) in POFs, and fluorescence microscopy on gold surface. The optimized SAM composition enabled the immobilization of about 112 ng/cm^2^ of aptamer. The thrombin detection exploiting POF-Aptasensor occurred in short times (5–10 minutes), the reached Limit of Detection (LOD) was about 1 nM, and the detection range was 1.6–60 nM, indicating the POF-Aptasensor well addresses the needs for a low-cost, simple to use and to realize, rapid, small size and portable diagnostic platform.

## Introduction

Developing rapid, cheap and sensitive methods for the determination of clinical biomarkers is of paramount importance in medicine, allowing the diagnosis, the prognosis and the choice of the therapeutic intervention. In this framework, sensing technology has been playing a key role in clinical practice^1^ in particular, opto-sensors have been offering advantages such as the label free determination of the analyte, fast responses and low detection limits^2^. Surface plasmon resonance (SPR) is among the most widely exploited sensing principle for the real-time monitoring of clinically relevant biomarkers^3,4^. The selectivity of the SPR measure is achieved by placing on the metal surface a specific receptor such as proteins^5^, molecularly imprinted polymers^6^, or aptamers^7^. The functional interface is key for the performance of the sensor, thus different chemistries can be exploited to couple the receptor to the metal^8^. In terms of optical setups, most SPR instruments rely on Kretschmann and Otto configurations^3^, whereas more recently optical fibers and gratings have been exploited offering reduced costs, miniaturization, remote interrogation and *in situ* application^9^.

In the present work, we developed an SPR sensing platform for the determination of thrombin (THR), clinical marker of the blood coagulation cascade and homeostasis^10^, based on a D-shaped plastic optical fibre (POFs)^9^, that provides ease of manipulation, simple fabrication, high sensitivity and competitive costs. The SPR-POF sensor was prepared by removing the cladding from the POF, spin coating a photoresist layer onto the exposed core and finally sputtering a thin film of gold of about 60 nm thickness^9^. The analyte selectivity of the POF-sensing platform depends on the specific receptor coupled to the gold surface:^11–13^ here a THR-specific DNA-aptamer was used. Aptamers are single-stranded nucleic acids offering the advantages of being easily modified with a variety of functional groups suitable for the coupling, of high stability even in non-physiological conditions and, unlike antibodies, the possibility to be regenerated, making the platform reusable^14,15^.

The selective aptasensing interface was prepared by modifying the gold with a Poly Ethylene Glycol (PEG) Self Assembled Monolayer (SAM)^16^ so to provide the highest accessibility for the analyte to the aptamer immobilized at the plasmonic surface, while decreasing the non specific adsorption. The PEG-SAMs are known to decrease the non specific protein adsorption, given their “brush” conformation, that is dependent both on the PEG chain density and on the molecular weight^17,18^. PEG-SAMs have been demonstrated to be effective of limiting the deposition of interferents at the plasmonic interface and in avoiding the consequent changes in the refractive-index that impair the sensing performance^19^. PEG and PEG-mixed SAMs have been reported to enable the determination of the biological markers straight from complex matrixes, such as blood, plasma or serum^19–22^. Furthermore, PEG-mix SAMs have been exploited for their ability to work in aqueous conditions^23–27^, thus being ideal for protein biomarker determinations, such as the case of THR. In the literature, both long^17,28^ and short PEG-SAM chains^29–31^ are reported. Moreover when mixed PEG-SAMs are used, a better surface control in terms of chemical reactivity has been observed^16,32,33^.

Here we planned to form the SAM by using PEGs functionalized with biotin so to exploit their streptavidin affinity for the further immobilization of a commercial biotin-modified THR-aptamer. We choose in particular the short PEG chains (C8), so to confine the thickness of the functional layer inside the evanescent field while maintaining non fouling properties^18,34,35^. A thiolated-PEG, a 6,8-dithio-octanoic-PEG and a biotinylated-PEG (Table 1) were mixed in different proportions with the aim to optimize the interface.

**Table 1:** Reagents for SAM preparation with the chemical formula reported and the molecular weight.

Name	Chemical structure	Molecular weight [Da]
PEGthiol		400.53
PEGlipo		571.79
BiotinPEGlipo		634.91

Next, the THR binding aptamer (TBA) was coupled to the PEG-SAMs through the avidin-biotin chemistry and the obtained functional interfaces were physico-chemically characterized. The performance of the TBA-POF for the detection of THR was evaluated, showing a nanomolar range of response and short analysis time (5–10 minutes). The preliminary results achieved so far suggested a promising TBA-aptasensor-POF that paves the way for the development of a low-cost and portable biosensor for the thrombin detection on site.

## Results

### Preparation and physicochemical characterization of the SAM interfaces

**Figure 1:**
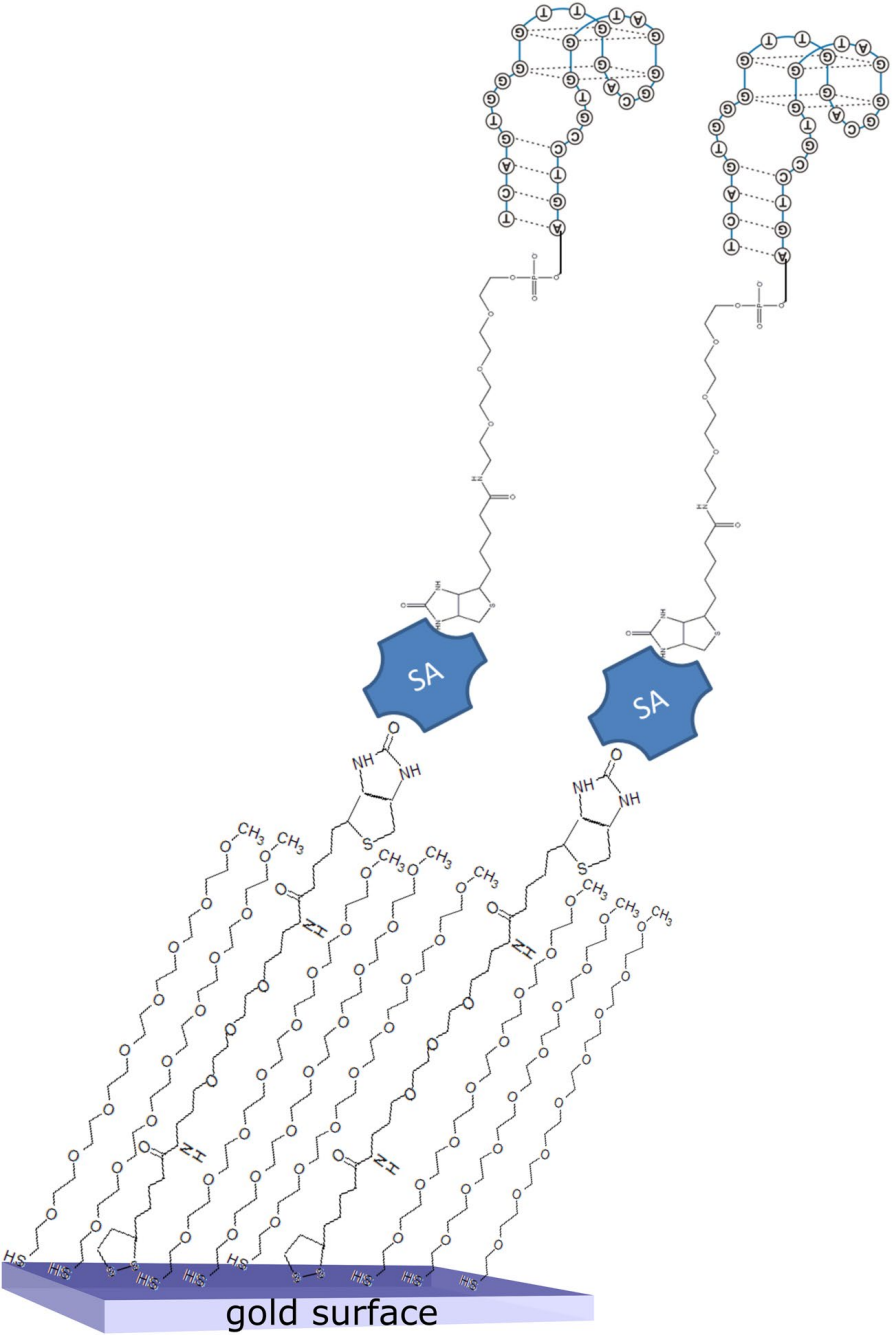
Scheme of the functionalization process. A biotin functionalized reagent is inserted in the SAM composition; streptavidin layer is immobilized on it and finally a biotin modified DNA-aptamer is attached.

SAMs were prepared according to the scheme reported in Fig. 1. The protocol for SAM formation was optimized on flat gold surfaces, intended as a model for the gold-POF platforms.

PEGthiol, PEGlipo and biotinylated-PEG-lipo reagents (formulas in Table 1) were used alone or at different molar ratio. XPS analysis and contact angle were used to confirm the derivatization steps (described in SI)^36,37^. The relative elemental composition and contact angle measurements are reported in Table 2.

**Table 2:** Chemical characterization determined by XPS analysis and water contact angles on different SAM on gold.

samples	O 1s (%)	C 1s (%)	S 2p (%)	Au 4f (%)	N 1s (%)	Thickness [nm]	Contact angle [°]
Au	18.0	17.6	3.1	57.5	3.7	—	<5
SAM (PEGthiol)	23.7	42.7	2.0	31.6	—	1.23	38.8 ± 2.8
SAM (PEGthiol + BiotinPEGlipo)	19.0	45.5	2.2	29.1	4.1	1.14	34.5 ± 1.9
SAM (PEGlipo)	17.5	42.2	2.3	35.6	2.5	0.66	35.1 ± 3.9
SAM (PEGlipo + BiotinPEGlipo)	18.1	42.1	2.3	34.3	3.2	0.65	37.5 ± 2.9

Mixed SAM are obtained with a PEGthiol or PEGlipo:BiotinPEGlipo with 8:2 molar ratio. XPS standard error does not exceed the 1–2% of the reported value.

The chemical composition of gold surface after argon plasma cleaning was in agreement with literature^18,28^, but showed sulphur and nitrogen contamination, disappeared after an overnight incubation in ultrapure water (Table S1 in SI). The SAM formation was indicated by a high carbon increase and with the decrease of the gold signal (see Table 2). The detailed chemical analysis performed collecting the core lines of the elements is reported in Fig. S1 (SI). The energy calibration was performed using the C1s and fitting the curve with two or three components depending on the sample. The first component related to the C-C/C-H bonds was set at 285.0 eV for all samples. The carbon on cleaned gold surface (“Au”) was mainly due to contaminations. For SAMs, the highest contribution was related to PEG chains (C-O) with binding energy between 286.7–287.1 eV in good agreement with the literature^28,30,38,39^. For mixed PEGthiol, PEGlipo or BiotinPEGlipo, an additional contribute due to C=O bonds at around 288.3–288.7 eV was detected, (according to the chemical formulation, Table 1). The oxygen peak was fitted with two components: the first one at 533.1–533.2 eV related to O-C bond in the PEG chains and the second one at 531.4–531.6 eV related to O=C bond^30^. The nitrogen signal appeared for BiotinPEGlipo and PEGlipo. There was one single component centered at 400.0–400.2 eV related to the amide bond^29^. Sulphur, mainly bound to gold, was indicated by the doublet at 162.2–162.6 eV while another component at 163.7–164.0 eV indicated the presence of unbound thiols^17,28,31^.

By using the attenuation in the gold signal it was furthermore possible to determine the SAM thickness^28^, whose values were reported in Table 2. The thickness measurement resulted in agreement with Ray *et al*.^29^ that used PEGs of similar molecular weight finding a value of about 1.3 nm (from XPS analysis) and Rafati *et al*.^31^ that estimated a thickness of about 1.7 nm. Al-Ani *et al*.^28^ calculated a thickness of 1–2 nm using longer PEG chains. Here the thickness values estimated for the PEGlipo SAM respect to the PEGthiol SAM might suggest a scattered orientation of the brushes on the surface, resulting in a lower accessibility of the biotin above the SAM (as indicated by the fluorescence measurement reported below).

The static contact angle measurements confirmed the gold substrate modification, showing an increment in the angle value from <5° (Au after plasma cleaning) up to about 35° for SAMs prepared (Table 2). Data were in agreement with^18,28^ that found a value of 43° for similar surfaces.

**Figure 2:**
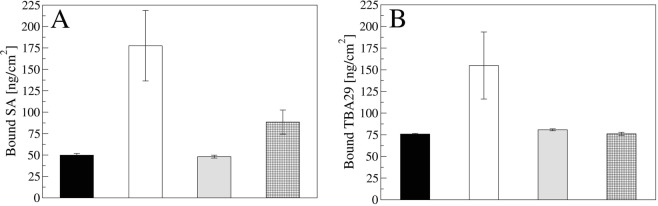
Immobilization of Cy3-Streptavidin on SAMs prepared with the following reagents: PEGthiol (black bar), PEGlipo (grey bar), PEGthiol:BiotinPEGlipo (white bar) or PEGlipo:BiotinPEGlipo (squared bar) in a 8:2 molar ratio (**A**). Immobilization of AlexaFluor488-labelled TBA29 aptamer on streptavidin immobilized on the same (**B**). Data are reported as mean value of three experiments acquiring 5 images for each sample. Error bars represent the standard deviation.

As an independent method to confirm the SAM formation, an indirect measure of the SAM was achieved using a fluorescent streptavidin (Cy3-labelled streptavidin). Figure 2A reports the adsorption of Cy3-labelled streptavidin to the SAMs prepared and expressed in ng/cm^2^ as calculated using calibration curve (Fig. S2 in SI). In agreement with expectations, only when the biotinylated reagent was present in the SAM mixture, a streptavidin immobilization was observed.

The optimization of the number of biotin baits on the surface was performed maintaining the total amount of reagents at 0.2 mM but varying the molar ratios of PEGthiol, PEGlipo and BiotinPEGlipo (Fig. S3). The highest number of biotin baits was observed for the mix PEGthiol:BiotinPEGlipo respect to PEGlipo:BiotinPEGlipo; presumably the orientation on the surface of the PEGthiol favored the accessibility to the biotin baits (Fig. S3), in agreement with what reported earlier for derivatization mixed PEG-SAMs^33^.

In the mixed SAMs (PEGlipo/BiotinPEGlipo and PEGthiol/BiotinPEGlipo) it was noticed an increase in the streptavidin immobilization at 9:1 molar ratio, whereas an additional increase of BiotinPEGlipo up to 6:4 molar ratio did not produce a significant increment in the fluorescence signal. Therefore, we selected BiotinPEGlipo at 8:2 molar ratio to form the mixed SAM as the optimal condition. When the BiotinPEGlipo quantity was further increased, the correlated fluorescence signal was consequently higher, as expected, but the homogeneity of the derivatized surface was lost (Fig. S4).

Next, we studied the conditions to immobilize the aptamer onto the optimized PEG-mix SAM surface (PEGthiol/BiotinPEGlipo 8:2 mol:mol). The PEG-mix SAM surfaces were then incubated with a fluorescent streptavidin (Cy3-SA) over time, in order to define the best immobilization time. A 5 μg/ml Cy3-SA solution was incubated on the SAMs and the fluorescence was monitored over time. Sixty minutes were set as optimal time incubation (Fig. S5). The bound Cy3-SA expressed in ng/cm^2^ as calculated using calibration curves (Fig. S2 in SI) is reported in Fig. 2A with the error bars (each experimental value is the average of three experiments acquiring 5 images for each sample and the error bar represents the standard deviation).

As a control, a fluorescent biotin-modified DNA-aptamer sequence (AlexaFluor488-TBA29) was incubated with the PEG-mix SAMs so to confirm that the immobilization of the biotinylated-aptamer occurred in a streptavidin-specific manner (Fig. 2B; calibration curve in Fig. S6 in SI). Results indicated the highest level of aptamer immobilization resulted for the PEGthiol/BiotinPEGlipo 8:2 mol:mol SAM.

### Preparation and physical characterization of the SPR-POF aptasensor

**Figure 3:**
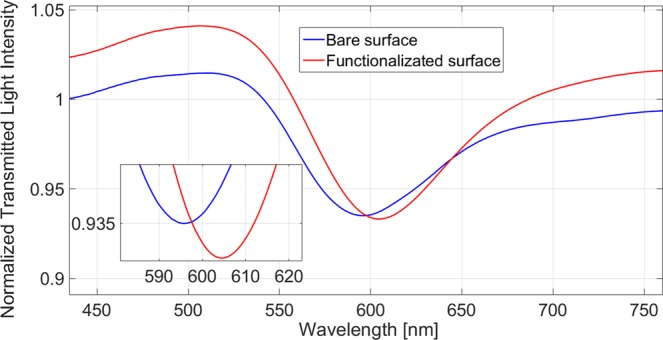
Resonance spectra, acquired in buffer solution as bulk, obtained before and after the functionalization process.

The optimized SAM protocol was transferred to the optical platform (Fig. S7 in SI). The quality of the gold layer on the SPR-POF platform was checked using XPS analysis. A proper gold amount, about 75–78%, was measured after plasma cleaning (Table S2; Fig. S8). The static contact angle measured on the POF after SAM formation (PEGthiol:BiotinPEGlipo 8:2 molar ratio) was equal to 34.4 ± 2.3°, in agreement with SAM measured on flat gold surfaces (see Table 2). The concentration of Cy3-labelled streptavidin immobilized on SAM on SPR-POF platform was 230.3 ± 6.3 ng/cm^2^, while the aptamer concentration was estimated 112.0 ± 2.9 ng/cm^2^ (using the calibration curves reported in the SI). The formation of SAM over the gold surface of the SPR-POF platform was also optically confirmed by the spectral curves. Figure 3 shows the normalized SPR transmission spectra (normalized to the reference spectrum) before and after the functionalization process, acquired in the presence of binding buffer as bulk. Both measurements were obtained by dropping about 70 μL of buffer solution over the planar sensing surface. In the presence of the same bulk medium (same refractive index, RI), before and after the functionalization procedure, a shift in the SPR transmission spectrum was observed indicating the resonance wavelength increased. The observed red-shift of ~10 nm, hinted at an increment in the RI at the gold surface, supporting the formation of the SAM.

### Detection of thrombin with the SPR-POF aptasensor

**Figure 4:**
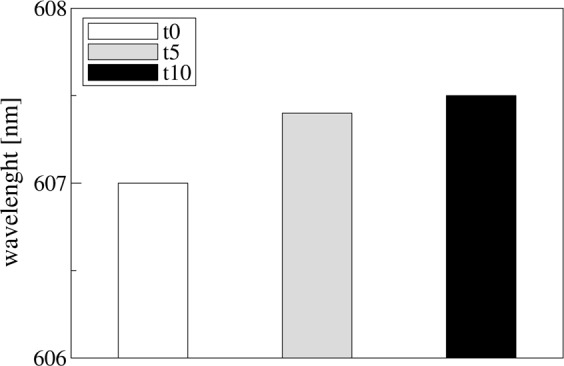
Wavelength measurements after addition of 20 nM of thrombin as function of time. Spectrum was acquired immediately after protein addition (white bar), after 5 minutes (grey bar) and after 10 minutes (black bar).

Figure S7 shows the used SPR-POF biosensor system. The kinetics of binding for THR on the SPR-POF aptasensor was studied. Transmission spectra were acquired over time: just after THR addition (t = 0), at 5 and at 10 minutes of incubation. Figure 4 reported the results obtained as function of time, showing that the 99% of the wavelength shift occurred within 5 minutes of incubation. The sensor response was fast when compared to other THR aptasensor, where the measurements were reported taking 20 to 60 minutes^40^.

**Figure 5:**
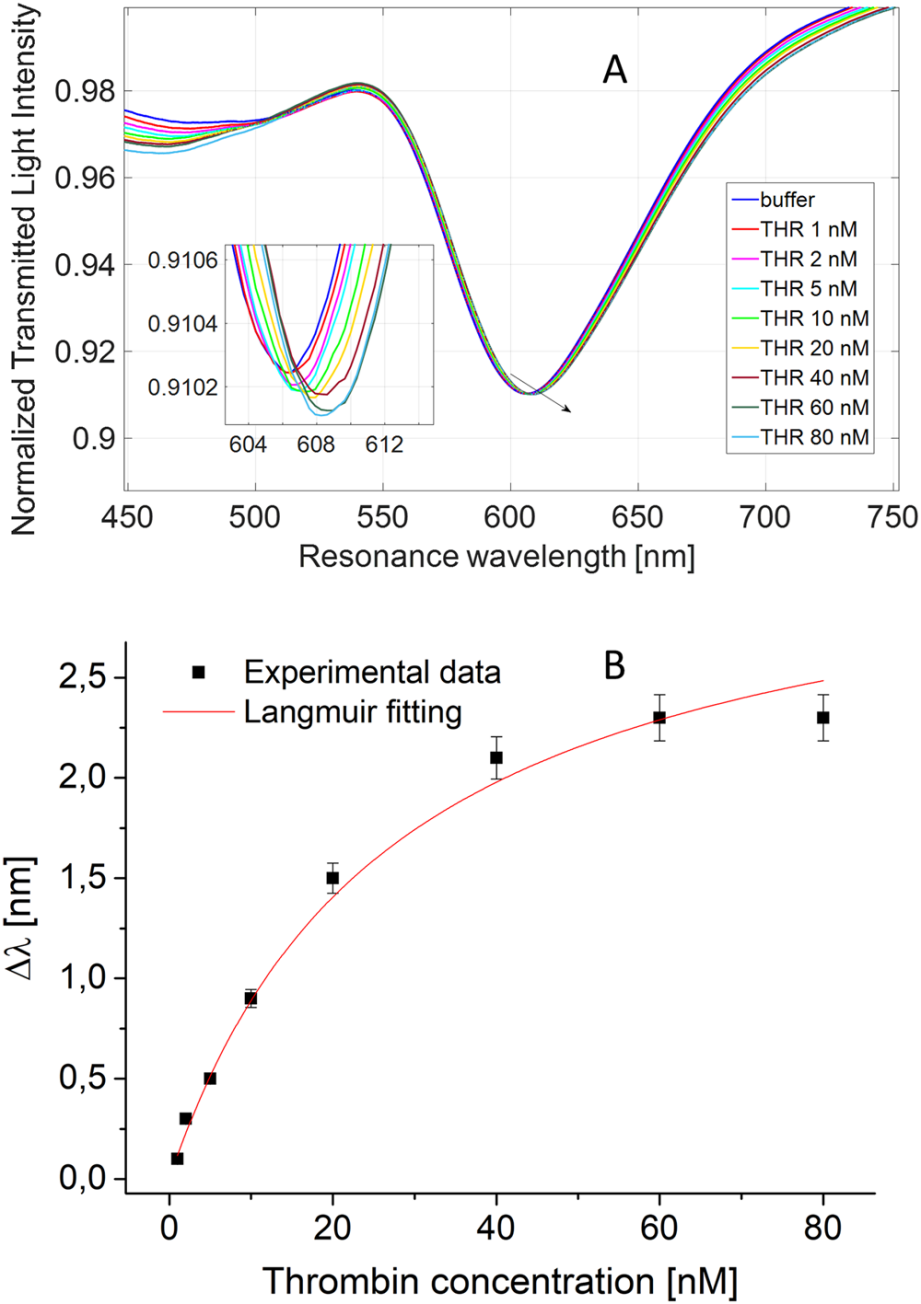
(**A**) SPR transmission spectra, normalized to the spectrum in air, for different concentrations of thrombin (1–80 nM). Inset: zoom of the resonance wavelengths region. (**B**) Plasmon resonance wavelength variation (Δλ), with respect to the blank, versus the concentration of THR (nM), with the Langmuir fitting to the experimental values and the error bars. Each experimental value is the average of 3 measurements obtained with three similar SPR-aptasensors (three similar SPR-POF platforms functionalized with the same bio-receptor); standard deviation (error bars) was within 5%.

The binding isotherm for THR on the SPR-POF aptasensor was devised, using THR concentrations in the range 1–80 nM. Figure 5 shows the SPR transmission spectra for THR (Fig. 5A). The resonance wavelength shift, with respect to the blank (buffer without the analyte), versus THR concentration was reported in Fig. 5B with the Langmuir fitting of data and the error bars (each experimental value is the average of 3 measurements, obtained with three similar SPR-aptasensors, and the error bar represents the standard deviation). A noticeable red shift occurred to the resonance wavelength, for increasing concentrations of THR, indicating the increment of the refractive index of the bio-layer in contact with the gold film. In support, the binding of THR to the functional interface was confirmed by an immunochemiluminescence experiment on flat gold surface (Fig. S9).

The dose-response curve, reported in Fig. 5B, were fitted by Langmuir equation:

1 $$\Delta \,{\rm{\lambda }}={{\rm{\lambda }}}_{{\rm{c}}}-{{\rm{\lambda }}}_{0}=\Delta \,{\rm{\lambda }}\,{\rm{\max }}\,\cdot \,({\rm{c}}/({\rm{K}}+{\rm{c}}))$$

where c is the analyte concentration, λ_c_ is the resonance wavelength at the concentration c, λ_0_ is the resonance wavelength at zero concentration (blank), Δλmax is the maximum value of Δλ (calculated by the saturation value minus the blank value). The parameters associated to the THR to aptasensor interaction were listed in Table 3 (values obtained by OriginPro8.5, Origin Lab. Corp. (Northampton, MA, USA)). As shown in Eq. (1), at low concentration of the analyte (c), i.e. much lower than K, the equation is linear, with sensitivity (slope) Δλ_max_/K, defined as the “*sensitivity at low concentration*”. The Limit of Detection (LOD) was calculated by the ratio of three times the standard deviation of the blank (stand. dev. of λ_0_, equal to 0.07 nm) and the sensitivity at low concentration (Δλ_max_/K, equal to 0.14 nm/nM) and resulted 1.6 nM.

**Table 3:** Parameters of the Langmuir equation relative to THR detection using 10 µM TBA-aptamer concentration.

Δλ_max_ [nm]	K [nM]	Statistics	
		Red. Chi-Sqr	Red. R-Square
3.07 ± 0.15	21.84 ± 2.8	0.007	0.99

The good performances of our aptamer platform were also confirmed when compared to the results of the direct immobilization of the thiol-aptamer on gold, previously reported^13^. The recognition of THR herein presented resulted >65 times more efficient respect to the interface prepared by direct immobilization of thiol-aptamer on the gold-POF. Such behavior could be explained by the better accessibility of the aptameric sequences to the analyte thanks to the herein designed PEG-based SAM interface (Fig. S10).

In order to verify the effect of the aptamer concentration on the sensor response, a SPR-POF aptasensor was prepared with a 10 times increase in TBA concentration (from 10 µM to 100 µM). The binding isotherm for the 100 μM TBA-aptasensor (Fig. S11; Tables S3 and S4) showed no significant improvement in the LOD and Sensitivity respect to the aptasensor prepared with low aptamer concentration. This behavior can be explained considering the steric hindrance produced by a too high aptamer density; aptamers need to assume a proper 3D conformation to recognize the target and therefore they need to have enough space in their surroundings.

### Selectivity analysis of the SPR-POF aptasensor

**Figure 6:**
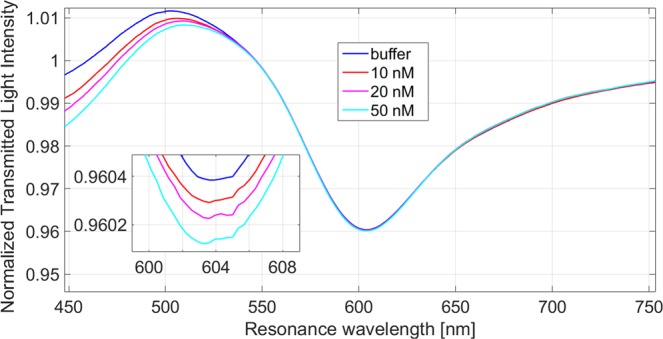
SPR transmission spectra for different concentrations of thrombin (1–50 nM) in the case of a reference sensor, functionalized with a non specific aptamer for THR.

The effects of the non specific binding was also tested, using a platform functionalized with a non specific aptamer sequence. When an aptamer non specific for THR was immobilized on the sensor, no significant shifts of the resonance wavelength occur with different THR concentrations, confirming the specificity of our platform. As shown in Fig. 6, when different THR concentrations, from 1 nM to 50 nM, are in contact with a non specific aptamer sequence, the resonance wavelength doesn’t change.

In view of using the POF-aptasensor for the determination of THR in plasma, preliminary experiments with real matrices were performed on flat gold surfaces derivatized with TBA and using immunochemiluminescence protocol as detection systems (SI). The prelimitary results, reported in Fig. S9, showed that PEG-biotin-streptavidin TBA designed SAM was indeed suitable to selectively capture THR directly from plasma, therefore indicating the possibility to translate the measure of real samples on the aptasensor-POF.

## Conclusions

By combining a low-cost and portable aptasensor system, based on a POFs to a specific aptamer, we demonstrated the detection of thrombin in the nanomolar range, in similarity with other aptasensor devices^41–43^. The aptasensor-POF demonstrated a remarkable LOD of 1.6 nM, offering advantages in the detection sensitivity as compared to the state of art in the field^40,44^.

In particular, the time of analysis for the aptasensor-POF was just 10 minutes, demonstrating a significantly faster time of responses, when compared to literature^40,44^ and suggesting that our low-cost and portable system can be used for building a reliable point-of-care system. In fact, the used optical transducer is very simple to realize, small size, low-cost and it doesn’t necessarily require a flow-cell system.

Additionally, the bio-interface herein proposed, exploiting a mixed SAM based on PEG and PEG-biotin function thus prone to avidin-binding, not only had the advantage of being totally build in water, but more importantly it constitutes a general design to prepare interfaces at high accessibility for the analyte, being it suitable for the immobilization of any biotin-modified aptamer. Thus, the SAM protocol herein proposed is of general value and can be exploited to the preparation of multiple POF-sensing platforms and sensing arrays for clinical needs.

## Materials and Methods

### Materials

DNA-aptamer sequence specific for Thrombin (5′-BiotinTEG-AG TCC GTG GTA GGG CAG GTT GGG GTG ACT-3′; 5′-BiotinTEG-AG TCC GTG GTA GGG CAG GTT GGG GTG ACT-AF488-3′) and the non specific sequence (5′-BiotinTEG-CA CCA ATA TTT ACG TTC TAC TCT CCA ATA-3′) are HPLC purified and were purchased from IDT Integrated DNA technologies (Leuven, Belgium). Purified Thrombin (THR) (Bioultra, T9326), Streptavidin from *Streptomyces avidinii* (85878) and all powders for buffer solutions were purchased from Sigma-Aldrich s.r.l. (Milan, Italy). Cy3-Streptavidin was purchased from Zymed Laboratories (San Francisco, CA, USA). A 100 nm of gold evaporated on silicon wafer is purchased from Agar Scientific (United Kingdom) and is utilized to characterize the functionalization process. The reagents utilized for the SAM preparation (Table 1) were purchased from Stratech (United Kingdom).

### Optical sensor platform and experimental setup

The plasmonic plastic optical fibre sensor (SPR-POF sensor) is based on a D-shaped POF, with the exposed core covered by photoresist and gold layers^9^. The SPR-POF sensor platform is shown in Fig. S1a, where the final length of sensing region is about 1 cm and the size of the used POF is 980 μm of core (1 mm in diameter). In particular, the POFs are especially advantageous due to their excellent flexibility, easy manipulation, great numerical aperture, large diameter, and the fact that plastic is able to withstand smaller bend radii than glass.

The measurements can be carried out by a spectral mode configuration. Fig. S1b shows the setup of a first prototype arranged to measure the transmitted light spectrum, which is able to measure the presence of biomolecules. The sensor system includes a halogen lamp ((HL–2000–LL, Ocean Optics), illuminating the SPR biosensor, and a spectrometer (FLAME-S-VIS-NIR-ES, Ocean Optics). The employed halogen lamp exhibits a wavelength emission range from 360 nm to 1700 nm, while the spectrum analyzer detection range was from 300 nm to 1,000 nm.

### Surface functionalization protocol

The procedure adopted is illustrated in Fig. 1. The gold surface is cleaned using an Argon plasma at 6.8 W for two minutes. Then, it is immersed in a MilliQ water solution containing 0.2 mM of PEGthiol or PEGlipo, or PEGthiol + BiotinPEGlipo (or PEGlipo + BiotinPEGlipo) in different molar ratio at room temperature for an overnight incubation. After the SAM formation, samples are washed in ultrapure water and dried with a stream of nitrogen. A 5μg/ml streptavidin solution (both fluorescent or not) in phosphate buffer (10 mM phosphate buffer, 138 mM NaCl, 2,7 mM KCl, pH 7.4) is applied for one hour. After washing in buffer, the surface was incubated with 10 μM of biotin-TBA29 aptamer (previously heated at 95 °C for one minute and leaved on ice for 10 minutes to unfold the aptameric sequence) for three hours in the same phosphate buffer. After a washing step in the same buffer used for incubation, the surface was ready for the measurements. The same procedure was adopted for the functionalization of the SPR-POF platform.

### Surface characterization

The functionalization process on flat gold substrate was characterized using XPS, static contact angle and fluorescence measurements. Details of the methods are reported in SI. The immobilization of the aptamers sequence on the gold surface was confirmed using a fluorescent derivative sequence, that is the same of the specific one, with an AlexaFluor488 fluorophore at the 3′ end. The readouts for the quantitation of the aptamer immobilization was performed by microscopy (SI). Signal measured with the standard imaging system was quantified using the ImageJ software^45^.

### Sensing experiments exploiting the SPR-POF aptasensor

Sensing experiments on SPR-POF biosensor were performed dropping about 70 μL of binding buffer (Tris 50 mM, EDTA 1 mM, MgCl_2_1 mM, KCl 150 mM pH 7.4) on the sensing region (D-shaped area), with an increasing amount of thrombin in buffer (spanning a range between 1 and 80 nM of protein). After a first set of measurements as function of time, a fixed incubation time of 10 minutes was selected for all measurements before the spectrum acquisition. A washing step with a binding buffer without the analyte, after the bio-interaction, was carried out and the spectrum was recorded.

## Supplementary information

Supplementary information
