# Supplementary material for: D-shaped plastic optical fibre aptasensor for fast thrombin detection in nanomolar range
Source: Sci Rep. 2019 Dec 10;9:18740. doi: 10.1038/s41598-019-55248-x (PMC6904456; doi:10.1038/s41598-019-55248-x)
Supplement: Supplementary file 1 — Supplementary information [file 41598_2019_55248_MOESM1_ESM.docx]

**Supplementary Information**

**D-shaped plastic optical fibre aptasensor for fast thrombin detection in nanomolar range**

Nunzio Cennamo^a,$^, Laura Pasquardini^b,$^, Francesco Arcadio^a^, Lia E. Vanzetti^c^, Alessandra Maria Bossi^d,*^, Luigi Zeni^a, **^

^a^ Department of Engineering, University of Campania “L. Vanvitelli”, Via Roma 29, Aversa, Italy

^b^ Indivenire srl, Via Alla Cascata 56/C, 38123 Trento, Italy

^c^ Fondazione Bruno Kessler-CMM-MNF, Via Sommarive 18, Trento, Italy

^d^ Department of Biotechnology, University of Verona, Cà Vignal 1, Strada Le Grazie 15, 37134 Verona, Italy

*Corresponding author: [alessandramaria.bossi@univr.it](mailto:alessandramaria.bossi@univr.it)

**Corresponding author: [luigi.zeni@unicampania.it](mailto:luigi.zeni@unicampania.it)

^$^These authors contributed equally to the work

**Static contact angle**

The static contact angle is measured using a home-made system. For each measurement 2 μl of deionized water is placed on the substrate. The images are acquired with CMOS camera and analyzed by Drop-Analysis [37]. For each sample, image of 3 drops placed in different zones are taken. The results are reported as average value and the errors are estimated as the standard deviations.

**XPS measurement**

XPS measurements on flat samples were performed using a Kratos Axis Ultra DLD (Kratos Analytical Ltd, England) instrument equipped with a hemispherical analyzer and a monochromatic AlK_α_ (1486.6eV) X-ray source, in spectroscopy mode. The emission angle between the analyzer axis and the normal to the sample surface was 0°, corresponding to a sampling depth of approximately 10 nm [36]. For each sample O 1s, C 1s, N 1s, S 2p and Au 4f core lines were recorded. The quantification, reported as relative elemental percentage, was done by using the integrated area of the fitted core lines, after Shirley background subtraction, and by correcting for the atomic sensitivity factors. This procedure gives a semiquantitative analysis, which is useful for the chemical characterization of the surface at different modification steps. XPS measurement on SPR-POF platform was instead performed using a Scienta ESCA 200 (Scienta Omicron GmbH, Germany) instrument equipped with a hemispherical analyzer and a monochromatic AlK_α_ (1486.6 eV) X-ray source, in transmission mode, since this instrument allowed the sample analysis. The emission angles between the analyzer axis and the normal to the sample surface are set at 0°, corresponding to a sampling depth of approximately 10 nm.

The overlayer thickness (d_OL_) were calculated based on attenuation length of the Au 4f_7/2_ signal using the following equation:

d_OL_ = λ_OL_ ln (I^0^_Au_/I_Au_) (S1)

where I^0^_Au_ is the measured intensity of the Au 4f_7/2_ peak on a pristine gold sample, I_Au_ is the measured intensity of the Au 4f_7/2_ peak of gold sample exposed to the solution, and λ_OL_ is the attenuation length of Au 4f_7/2_ photoelectrons in the polymer overlayer (3.45 nm) [28].

**Table *S1*: Chemical characterization determined by XPS analysis on gold before and after incubation in water overnight. XPS standard error does not exceed the 1-2% of the reported value**

|  | **samples** | **O 1s (%)** | **C 1s (%)** | **S 2p (%)** | **Au 4f (%)** | **N 1s (%)** |
| --- | --- | --- | --- | --- | --- | --- |
| 2 | Au after plasma cleaning | 18.0 | 17.6 | 3.1 | 57.5 | 3.7 |
| 3 | Au after plasma cleaning and water incubation | 13.0 | 39.4 | - | 47.7 | - |


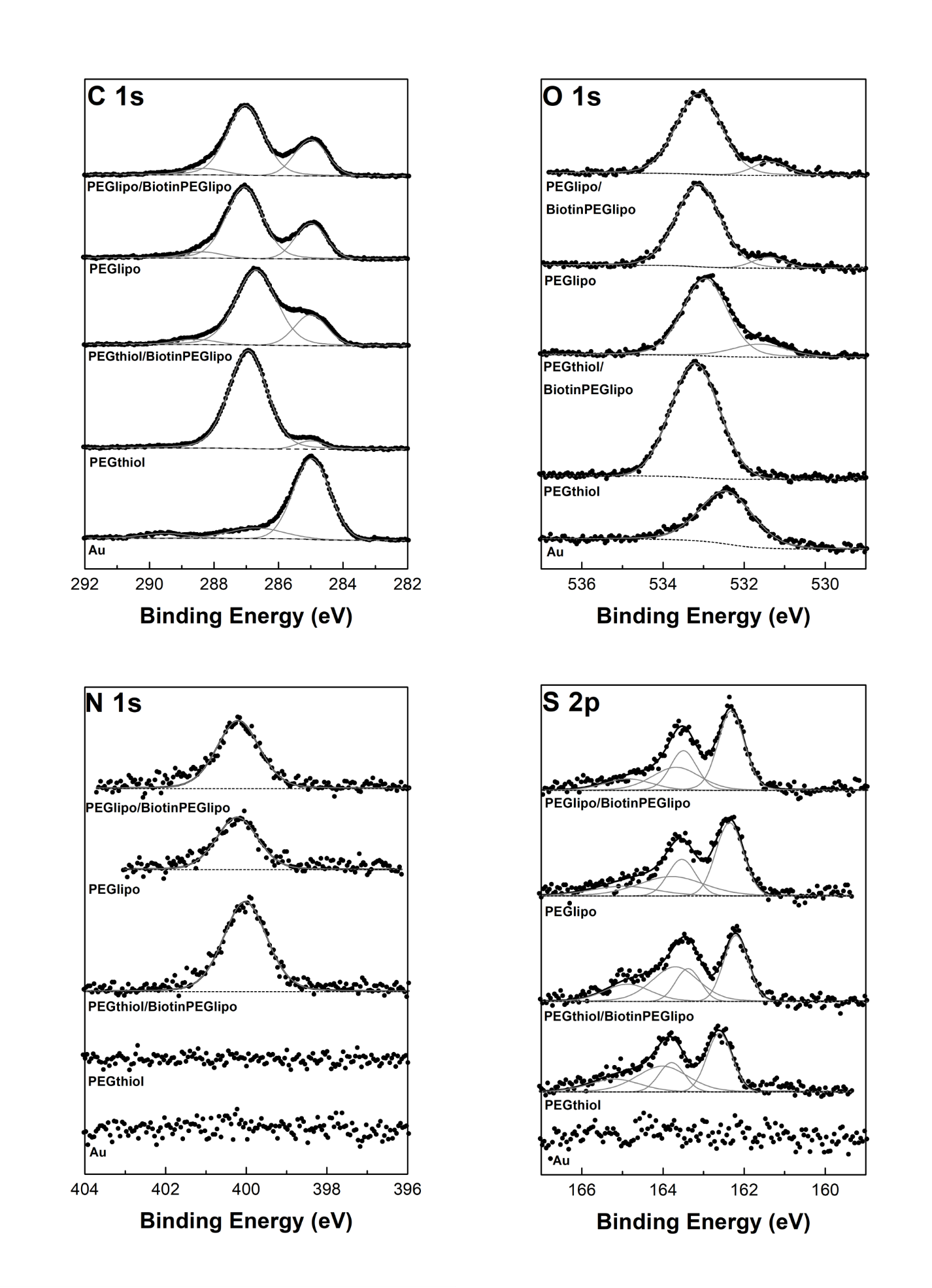


Figure S1: Detailed core lines for the different surfaces. “Au”: bare gold surface after plasma cleaning and water incubation overnight. Different SAM prepared with pure reagent (PEGthiol or PEGlipo) or mixed SAM prepared in a 8:2 molar ratio (PEGthiol/PEGlipo: BiotinPEGlipo). Main chemical components are indicated for each element and discussed in the main text.

COMPLEMENTARY MEASURES

**Fluorescence characterization**

The Cy3 fluorescence signal on streptavidin molecule was monitored using a fluorescence microscope (Leica DMLA; Leica Microsystems, Germany) equipped with a mercury lamp and the fluorescence filter N2.1 (Leica Microsystems, Germany), while the AF488 signal was detected using the fluorescence filter L5 (Leica Microsystems, Germany). All samples were observed with a 20X magnification objective and measured with a cooled CCD camera (DFC420C, Leica Microsystems, Germany). The signal was analyzed using the ImageJ software [45].

Calibration curves of fluorescent molecules were obtained immobilizing known amount of fluorescent molecules (both Cy3-labelled streptavidin and AlexaFluor488-labelled TBA29 aptamer) on flat gold surface. Once dried the fluorescent signal was acquired using the same instrumental parameters utilized for the experiments and a calibration curve was obtained. Calibration curves are reported in the Figure S2 and S6.


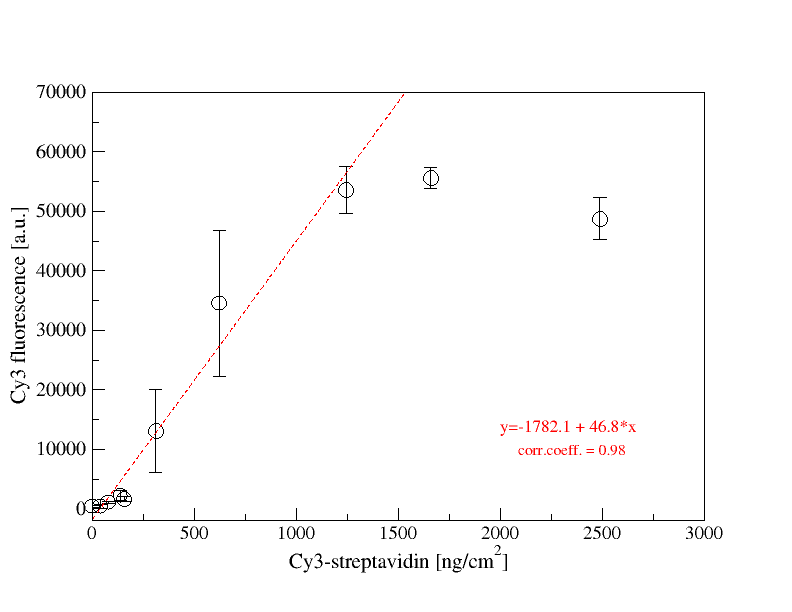


*Figure S2: Calibration curve of Cy3-labelled streptavidin. Data are reported as mean value on three images and error bars represent standard deviation. Red dashed line is the linear fit performed on 8 points.*


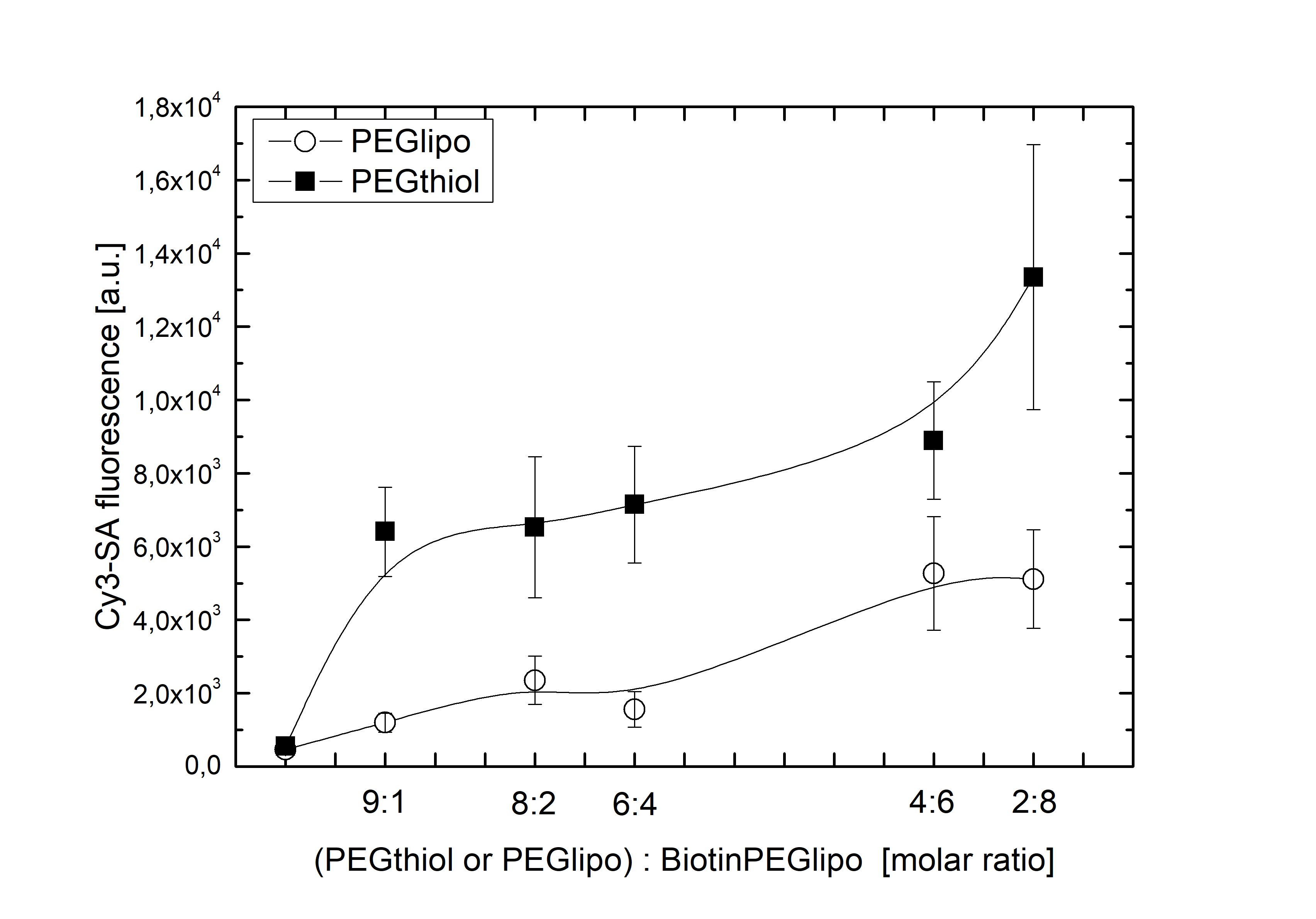


Figure S3: Cy3-Streptavidin on SAM prepared using different concentration of biotinylated reagent and the two reagents based on PEG molecule. Data are reported as mean value of two different experiments, acquiring 5 images for sample. Error bars represent the standard deviation.


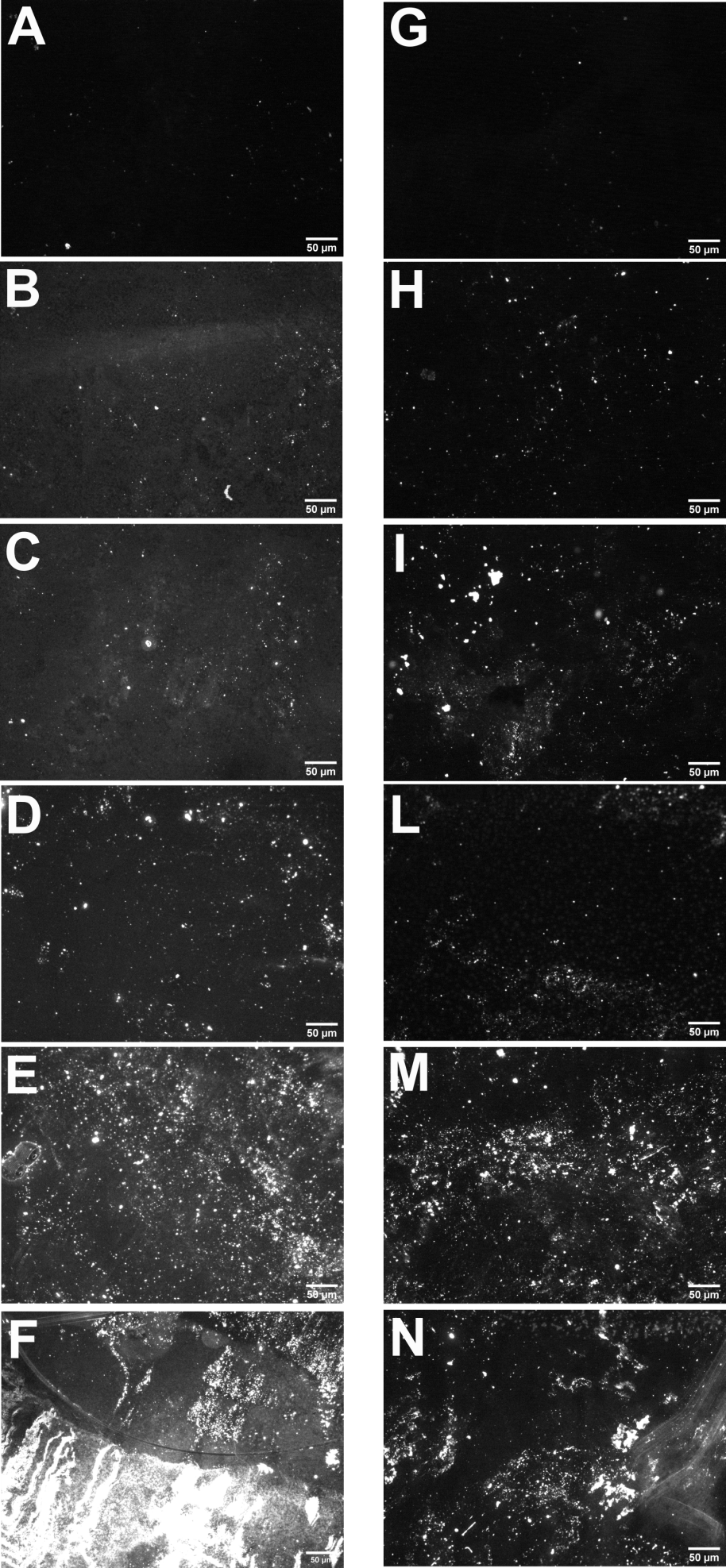


Figure S4: Fluorescent streptavidin on SAM with increasing concentration of BiotinPEGlipo on SAM based on PEGthiol (A-F) or PEGlipo (G-N). PEGs:BiotinPEGlipo molar ratio: (A-G) 0; (B-H) 9:1, (C-I) 8:2, (D-L) 6:4, (E-M) 4:6, (F-N) 2:8.


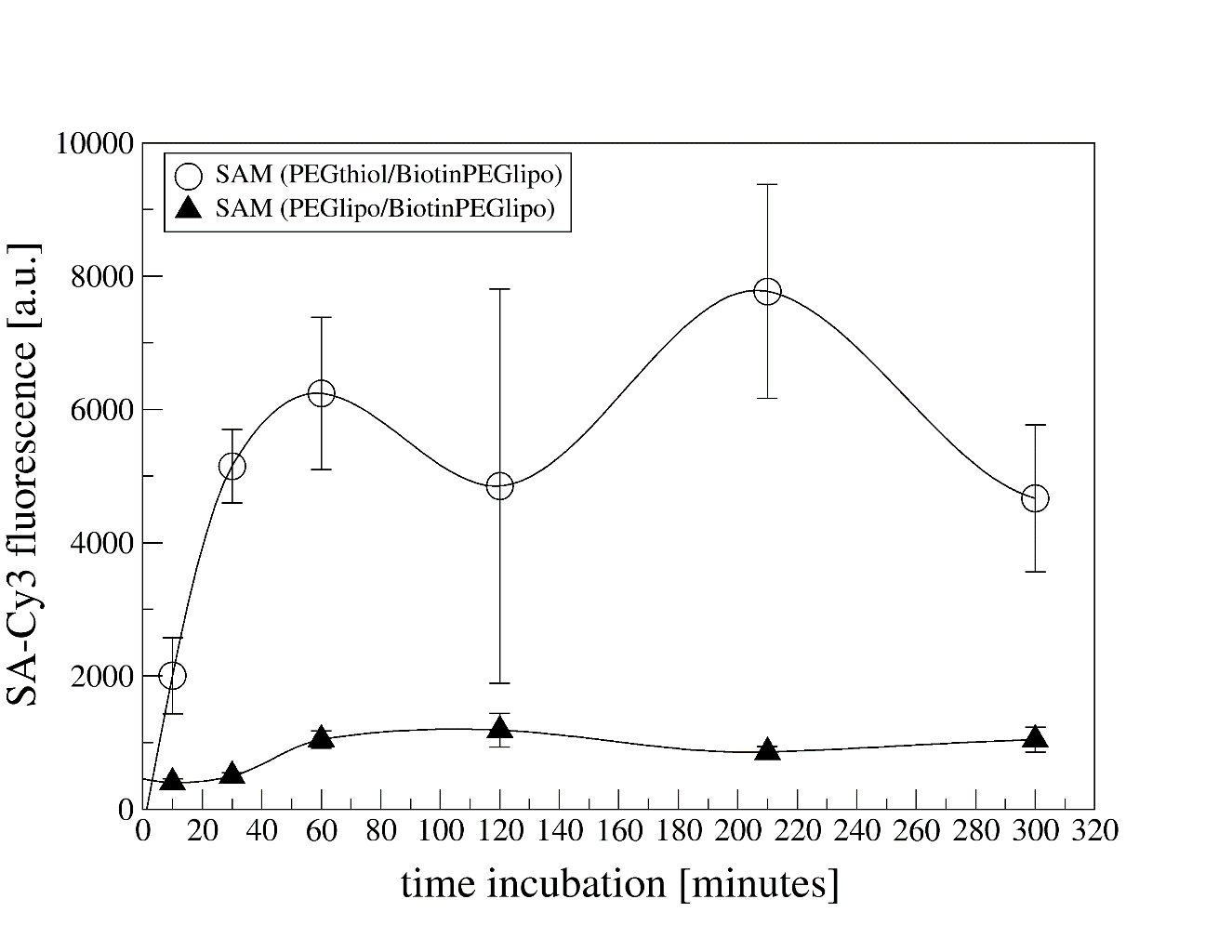


Figure S5: Fluorescent streptavidin at 5μg/ml concentration on SAM (8:2 molar ratio) at increasing time. Data are reported as mean value of 5 acquisitions on each sample and error bars represent the standard deviation.


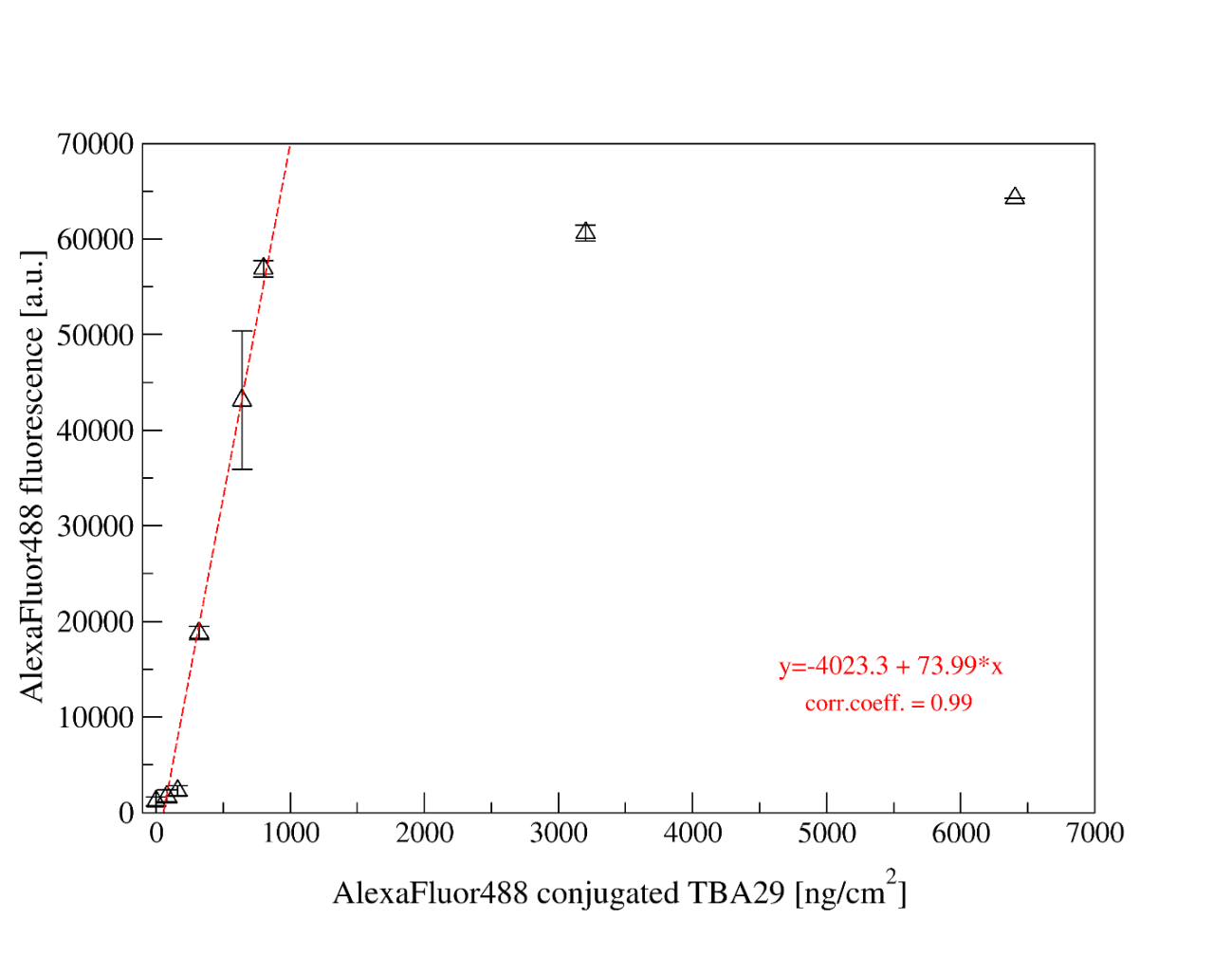


*Figure S6: Calibration curve of AlexaFluor488-labelled TBA29 aptamer. Data are reported as mean value on three images and error bars represent standard deviation. Red dashed line is the linear fit performed on 6 points.*

OPTICAL SETUP

Figure S7 reports the outline of the sensing SPR platform and the optical setup.


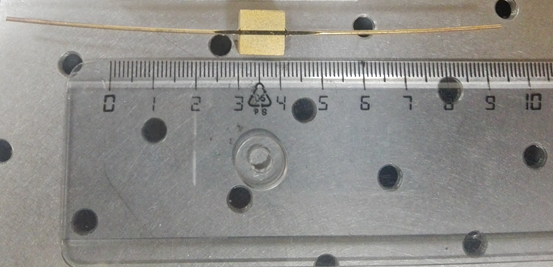


(a)


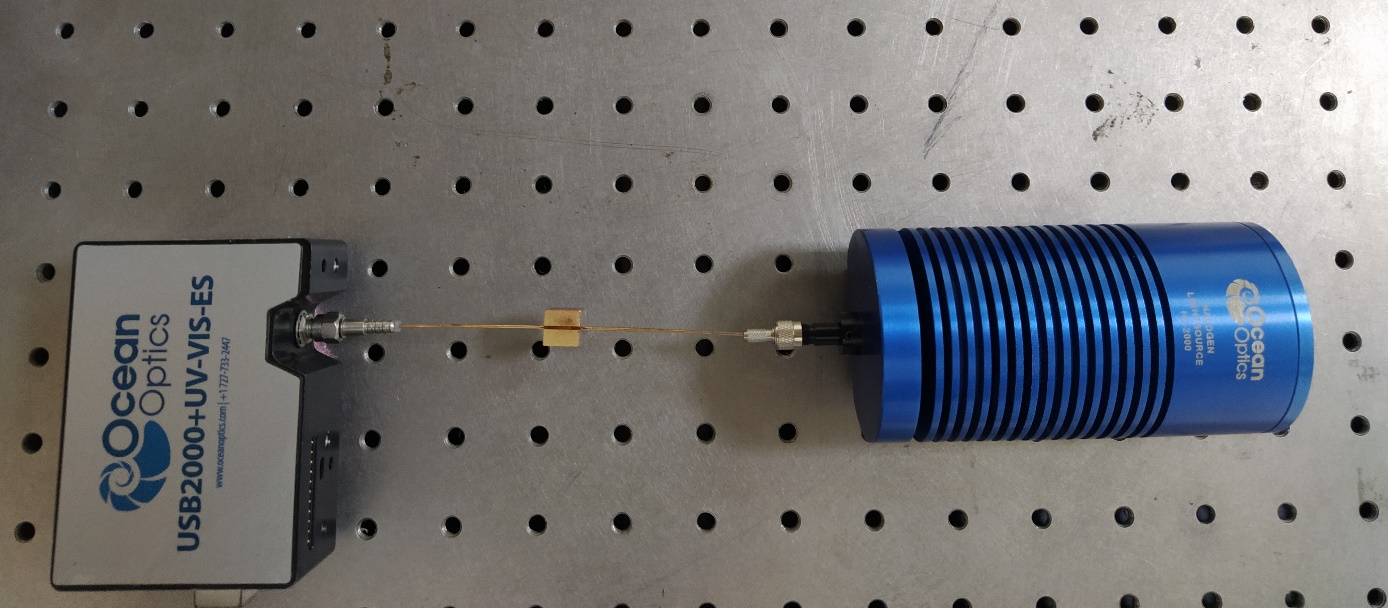


(b)

*Figure S7: (a) Picture of the SPR-POF platform; (b) Picture of the Sensor System*

Table S 2: Elemental composition determined by XPS analysis on SPR-POF platform (on fiber or out of fiber). The standard error does not exceed the 1-2% of the reported value.

| **Sample ID** | **O 1s (%)** | **C 1s (%)** | **Au 4f (%)** |
| --- | --- | --- | --- |
| **On fiber** | 3.6 | 17.8 | 78.6 |
| **Out of fiber** | 3.6 | 20.8 | 75.6 |


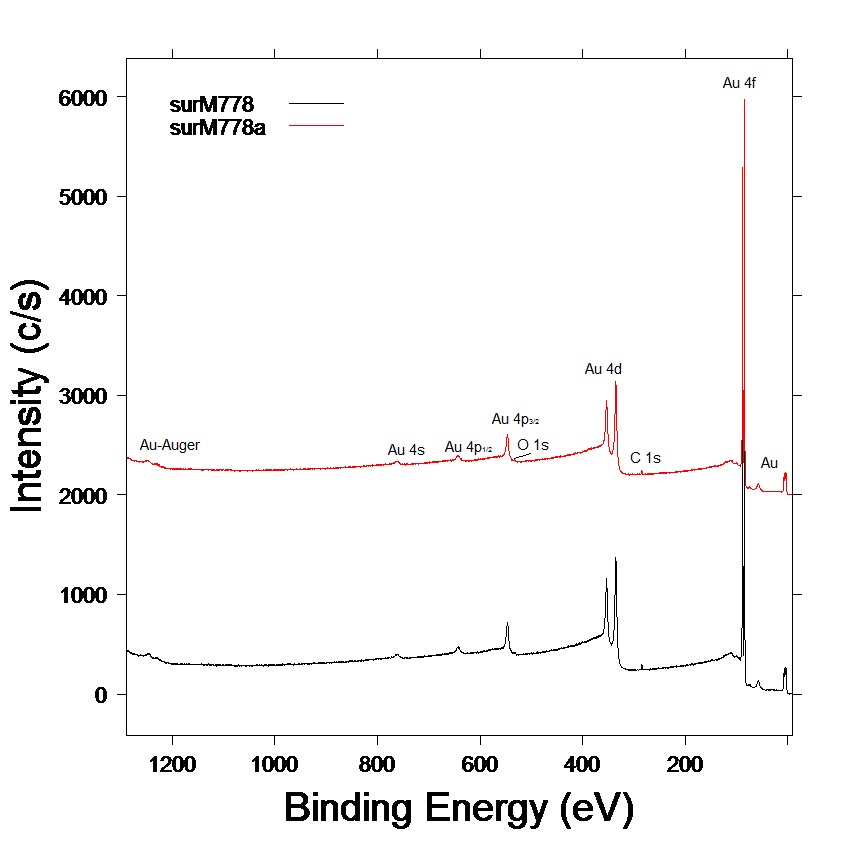


Figure S 8: Survey spectra on fiber covered by gold layer (red spectrum) or out of the fiber (black spectrum).

PROTEIN DETECTION ON GOLD SURFACE

The protocol developed for the THR detection on flat gold surface is based on an immunochemiluminescence method. The protein incubation is performed in buffer (Tris 50 mM, EDTA 1 mM, MgCl_2_1 mM, KCl 150 mM pH 7.4) for 1 h on a slow orbital shaker and followed by a washing step in the same buffer. Before proceeding with the protein detection, the surfaces are passivated in 3% w/v BSA for 30 min. A primary mouse anti-thrombin antibody at 2.5 μg/ml concentration in 3% w/v BSA is applied with an incubation of 30 min. The excess of antibodies is removed with three extensive washing steps. The samples are then incubated for 30 min with an antimouse HRP-conjugated secondary antibody at 2 μg/ml concentration in 3% w/v BSA. After the incubation, the surfaces are washed three times in buffer and the chemiluminescence signal was developed with the SuperSignal West Femto Chemiluminescent Substrate kit according to the manufacturer instructions, using a standard imaging system ChemDoc-It (Bio-Rad), acquiring the signal for 0.5 second. Signal measured with the standard imaging system was quantified using the ImageJ software [45].

The ability of the interface on gold in the thrombin recognition was assessed using a chemiluminescence protocol. The thrombin detection on our platform (TBA29 aptamer immobilized on PEGthiol:BiotinPEGlipo, 8/2 molar ratio) was performed in buffer solution on standard gold surface. Figure S9 reports the results obtained in a thrombin range between 1 and 200 nM and the relative Langmuir fitting performed in buffer or in complete human serum.





Figure S 9: *thrombin incubation on TBA29 aptamer immobilized on optimized SAM interface (PEGthiol/BiotinPEGlipo, 8/2 molar ratio) in buffer solution (black circles) or complete human serum (white triangles up). Data are reported as mean value of at least two different experiments and error bars represent the standard deviation.*

The performances of our platform were tested also in combination with another protocol based on the direct aptamer immobilization on gold (protocol used in our previous work [13]). A low signal of the previously used protocol respect to our SAM interface was recorded (more than 65 times lower), as reported in Figure S10.


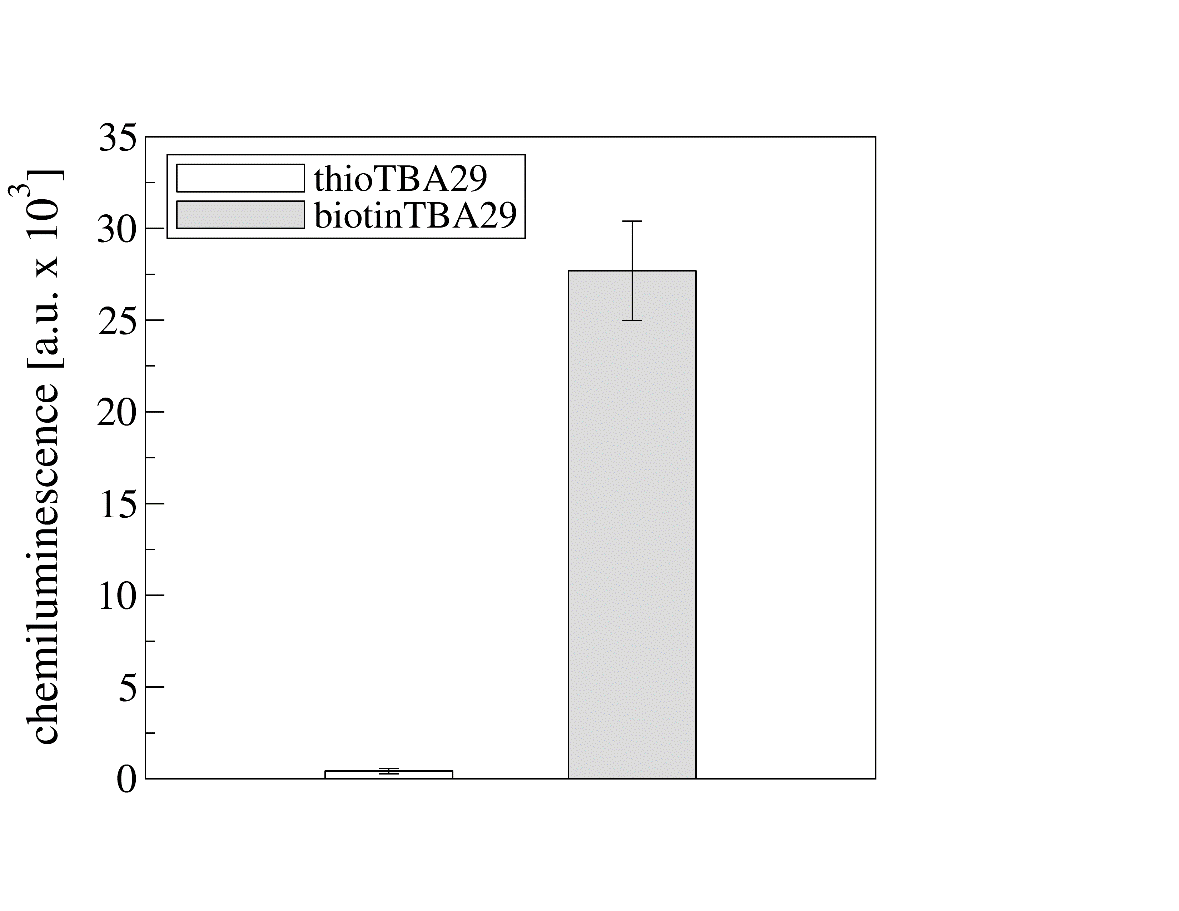


*Figure S 10: Immunochemiluminescence on 50 nM Thrombin incubated on aptamer layer prepared on our SAM interface (biotinTBA29) or on aptamer directly immobilized on gold (thioTBA29) using the protocol reported in Cennamo et al. [13].*

A CONTROL EXPERIMENT ON SPR-POF APTASENSOR


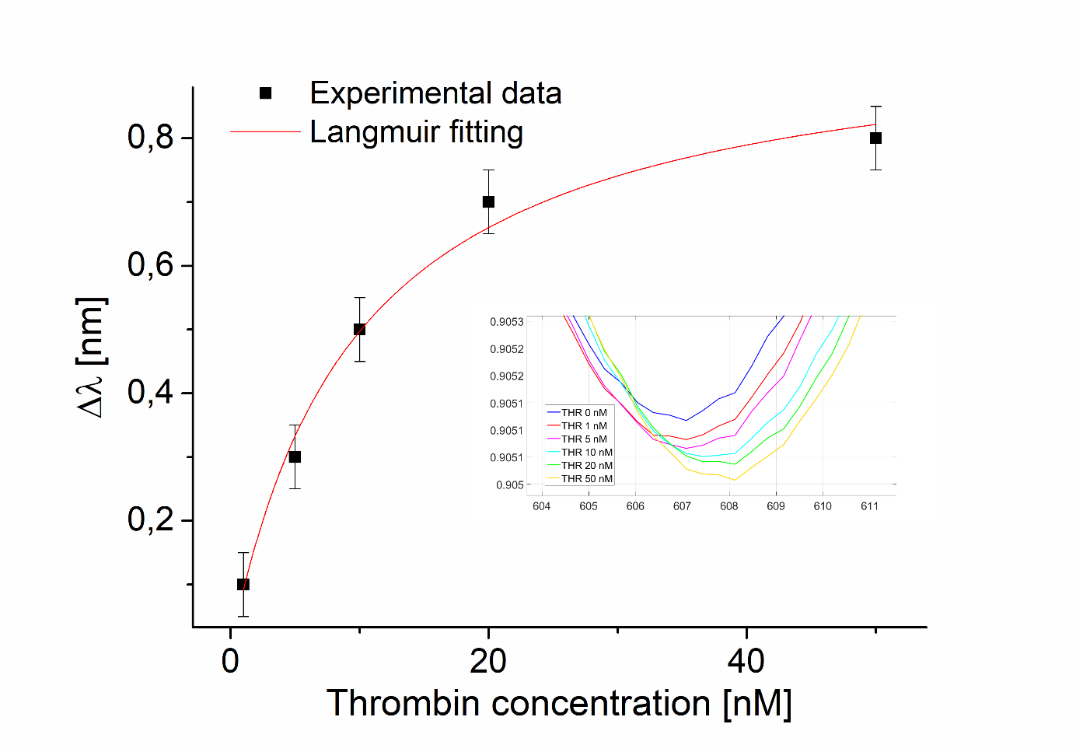


*Figure S 11: Plasmon resonance wavelength variation (Δλ), with respect to the blank, versus the concentration of THR (nM) and Langmuir fitting to the experimental values. Inset: zoom of the relative resonance wavelengths. Configuration with an SAM obtained by an Aptamer concentration of 100 µM.*

Table S 3: Langmuir Parameters using a TBA-aptamer concentration of 100 µM

| **Δλ_max_ [nm]** | **K [nM]** | **Statistics** | |
| --- | --- | --- | --- |
|  |  |  | |
| 0.98 ± 0.06 | 9.76 ± 1.6 | Red. Chi-Sqr | Red. R-Square |
|  |  |  |  |
|  |  | 0.01 | 0.99 |
|  |  |  |  |

| **Chemical Parameters** | |
| --- | --- |
|  | |
| Sensitivity at low c of THR  (Δλmax/K)  [nm/nM] | Limit of detection (LOD) [nM]  (3*standard deviation of blank (0.04 nm)  / sensitivity at low c of THR) |
|  | |
| 0.10 | 1.2 |

Table S 4: Chemical parameters obtained by Langmuir Parameters using a TBA-aptamer concentration of 100 µM
